# In Memoriam—P. P. Wells, M. D.

**Published:** 1891-12

**Authors:** Edmund J. Lee


### IN MEMORIAM—P. P. WELLS, M. D.

In announcing to the homœopathic medical profession the sad news of the death of Dr. Phineas Parkhurst Wells, we feel we chronicle the greatest loss to medicine since the deaths of Constantine Hering and Adolph Lippe. Another, almost the last, of the able men known as the "old guard," has left our ranks for good, and none can point out his successor! For it is not overstating the truth to assert that no homœopath, since Hahnemann, has done more to teach the true principles of Homœopathy than did our venerable friend and teacher, P. P. Wells. The object of his teaching was rather to inculcate correct doctrine than to teach the materia medica, as did Hering and Lippe. He believed, and correctly too, that each practitioner should be taught how to study and to apply the materia medica rather than to be taught the materia medica itself or its application to any special cases. No worthier or abler follower of Hahnemann has yet honored the ranks of the homœopathic school; he was indeed the compeer of any of the able men who were his associate practitioners for so many years, and fellow-pioneers in establishing Homœopathy in America.

As is well known to our readers, Dr. Wells had been an invalid for many years; for the last year or more he had been confined to his bed or his chair, weak in body but still vigorous in mind. Feeling that his work in this world was done, and well done too, this old warrior could well recline upon his couch and calmly await his release, with Christian fortitude and hopefulness. Like the Apostle St. Paul, he too could well exclaim: "I have fought the good fight, I have finished *my* course, I have kept the faith." And so with work completed, house in order, and folded hands, this veteran Christian physician departed this life, Monday morning, November 23d.

Dr. Wells was born in New Hampshire, in 1808, was first a printer, and later studied medicine, graduating at Dartmouth in 1833. About the year 1843, he moved to Brooklyn and began the practice of Homœopathy there. At the same date came also Dr. A. C. Hull. These two, as then required by law, applied for membership in the Allopathic County Medical Society, but being homœopaths were rejected. Dr. Hull brought suit against the society to force them to elect him a member, which suit he won after *sixteen* years. He then very properly refused to join them, as a homœopathic society had in the meanwhile been organized. Dr. Wells prospered in his practice, it increasing steadily as his energy, ability, and conscientious skill merited. For many years he was the leading homœopathic practitioner of Brooklyn. So devoted were his patients to him that they continued to call and consult him even while confined to his bed. He was most conscientious and diligent in his work and very successful as a healer.

Dr. Wells was a thorough believer in Homœopathy, in all of it. He did not accept part and reject part; he believed in the law of the Similars, in the single remedy, in the minimum dose of the potentized remedy; he believed in chronic miasms as causes of disease. He believed all these because he had tried them one by one, and had proven them to be true. At the meeting of the I. H. A., at Saratoga, Dr. Wells said, as his farewell words to his friends: "I have hardly words, Mr. President, to express my gratification at the approval of yourself and our associates, and the more because I am quite impressed with the probability that this is the last meeting of our Association I shall ever attend. The probabilities are that before you assemble again I shall be called up higher. I was not in favor originally of the formation of this Association. I thought my mission was rather in the old Institute, which I helped to create, and thought that there I should strive to bring it into a state of life and truthful activity, from which it has departed. I have changed my mind. I have given my whole interest and affection to this Association; and if I am never permitted to meet you again, I would like to leave with those who survive me my testimony, once and forever, to the truth of the law which governs our Association, which has our utmost confidence, and to urge the Association, if I am gone, to spare no effort, to count no exertion too much which shall extend the confidence we have in our law, and which shall increase our influence to induce others to come into active support of our truth."

In his address as President of this Association, he gives us the keynote of the success of his life. After a masterly analysis of the principles of homœopathic philosophy, he adds: "What, then, are the members of this Association to do, the results of which shall justify their existence as an associated body? We know of but one thing, and that is *work*—earnest, honest, incessant work." And it was just this kind of work that made Dr. Wells a leader in Homœopathy. He was not satisfied, as most physicians are, to labor only as a practitioner; not satisfied to be merely a skillful homœopathic physician, whereby he could gain fame and wealth, but he desired to lead others, to teach others the true practice. He labored not only for himself, but for the whole profession. So we find the chief homœopathic journals for the past forty years have been enriched by his pen. And no writer in all the field of homœopathic literature has written better, more forcibly, or more consistently than he, for during all those years Dr. Wells taught true Homœopathy.

He was very active in the meetings of many medical societies; was one of the few who organized the American Institute; also, later on, the International Hahnemannian Association. His address before that Association, at its first annual meeting, was a superb effort, outlining the character and purposes of the new organization. It might well be read at the opening of each annual meeting, to serve as a reminder of its original purpose, and as an incentive to energetic work.

Ever since THE HOMŒOPATHIC PHYSICIAN was established, Dr. Wells has been a steady and most valued contributor to its pages. Many of these papers are classics amongst homœopathic literature, which will amply repay frequent and careful study. It is to be hoped that the most useful of them may yet be gathered together in book form for permanent keeping.* Our school possesses no such essays from any other pen and hence cannot afford to allow these to be neglected and lost. Amongst the many and varied contributions to our current literature from this gifted pen may be mentioned essays upon rheumatism, scarlet fever, typhoid fever, diarrhœa, dysentery, and his last effort, now being published in this journal, a treatise upon the treatment of intermittent fever. This last is a masterpiece and is, as Dr. Wells himself said, the crowning work of his life.

The secret of Dr. Wells' success in practice and of his influence upon his colleagues may be found, we believe, in the advice already quoted, "earnest, honest, incessant work;" at another time he said: "I have been all my life a learner." An earnest, honest man influenced by a love of truth and for his fellow-men

---

* A list of the most useful and practical of these essays has been given in this journal, Volume XI, p. 51.

could not fail of success; it is a laborious, difficult path but a sure one.

The homœopathic school in America can ill afford to lose such a man as Dr. Wells; he leaves a void not readily filled. Yet if those who are left to take up and continue his work are influenced by the same honesty, the same energy and love of the truth, then the good work will not lag nor will we cease to be thankful for the life and example of our departed friend and teacher. EDMUND J. LEE.

